# Evaluating first-line therapeutic strategies for metastatic castration-resistant prostate cancer: a comprehensive network meta-analysis and systematic review

**DOI:** 10.3389/fonc.2024.1378993

**Published:** 2024-04-15

**Authors:** Duojie Zhang, Haimin Weng, Zhangji Zhu, Weilun Gong, Yinfeng Ma

**Affiliations:** ^1^ The Second Clinical Medical College of Zhejiang Chinese Medical University, Hangzhou, Zhejiang, China; ^2^ Department of Urology, The Second Affiliated Hospital of Zhejiang Chinese Medicine University, Hangzhou, Zhejiang, China

**Keywords:** castration resistant prostate cancer, first-line treatment, chemotherapy, antihormone therapy, network meta-analysis

## Abstract

**Objective:**

This study aimed to evaluate the relative efficacy and safety of first-line treatment options for metastatic castration-resistant prostate cancer (mCRPC).

**Methods:**

We systematically searched electronic databases, including PubMed and Web of Science, for studies published from their inception to April 3rd, 2023. Inclusion criteria were: 1) Completed Phase III or IV randomized controlled trials (RCTs) registered on ClinicalTrials.gov; 2) Patients with a confirmed diagnosis of mCRPC who had not previously received chemotherapy or novel endocrine therapies. We conducted a network meta-analysis using R software (version 3.4.0). Network graphs and risk of bias graphs were generated using Stata 14.0 and RevMan 5.4, respectively. The primary outcome was overall survival (OS), and the secondary outcome was the incidence of severe adverse events (SAEs).

**Results:**

Seven RCTs encompassing 6,641 patients were included. The network meta-analysis revealed that both docetaxel+prednisone (DP) and cabazitaxel+prednisone (CP) significantly improved OS compared to abiraterone. Compared to placebo, DP showed comparable results to both cabazitaxel 20 mg/m^^2^+prednisone (C20P) and cabazitaxel 25 mg/m^^2^+prednisone (C25P) in terms of OS. For SAEs, both DP and C20P were superior to C25P, with no statistical difference between C20P and DP. The probability ranking plots indicated that C25P ranked highest for OS, while DP ranked highest for SAEs.

**Conclusions:**

Based on our network meta-analysis, we recommend cabazitaxel 20 mg/m^^2^+prednisone (C20P) as the primary choice for first-line management of mCRPC, followed by DP. Enzalutamide and abiraterone are suggested as subsequent options. Radium-223 may be considered for patients presenting with bone metastases.

**Systematic review registration:**

https://www.crd.york.ac.uk/prospero/, identifier CRD42023443943.

## Introduction

Prostate cancer (PC), the most prevalent malignancy in the male genitourinary system, has recently emerged as the second most common cancer globally (1). The world age-standardized incidence rate is 37.5 per 100,000, with higher prevalence in regions with a high Human Development Index, such as Europe and North America. Many PC patients undergo Androgen Deprivation Therapy (ADT) post-laparoscopic or robotic surgery, showing promising efficacy in the initial and intermediate stages. However, due to various mechanisms such as androgen receptor amplification, mutation, PI3K pathway, or NF-κB pathway aberrations, tumors often develop resistance to ADT, progressing to mCRPC within 18-24 months, frequently accompanied by distant metastases (2). This phase is marked by a dismal prognosis and escalated treatment costs (3). Current therapeutic approaches include second-generation antiandrogens (e.g., abiraterone, enzalutamide, apalutamide), chemotherapy (docetaxel, cabazitaxel), and radionuclide therapy (Radium-223, 177Lu-PSMA) (4, 5). Abiraterone, a CYP17 inhibitor, diminishes androgen levels by inhibiting a crucial enzyme in androgen synthesis. Enzalutamide and apalutamide, as androgen receptor antagonists, prevent androgen from binding to its receptor. Clinical trials have demonstrated the effectiveness of abiraterone and enzalutamide in extending progression-free survival (PFS) and overall survival (OS) in mCRPC patients (6, 7).

Despite this, the absence of direct comparative trials for first-line treatments leaves a gap in knowledge regarding the optimal balance of efficacy and safety. This study aims to fill this void by comparing the effectiveness and safety of first-line mCRPC treatments as reported in randomized clinical trials (RCTs), thereby guiding clinical decision-making.

## Methods

### Eligibility criteria

The inclusion criteria for trials were as follows: 1) Phase III or IV randomized controlled trials (RCTs); 2) Participants diagnosed with metastatic castration-resistant prostate cancer (mCRPC); 3) No history of cytotoxic therapy or androgen receptor inhibitor therapy; 4) Interventions including abiraterone acetate, enzalutamide, apalutamide, docetaxel, cabazitaxel, or Radium-223; 5) Outcomes measuring overall survival (OS) and severe adverse events (SAEs). Exclusion criteria comprised: 1) Studies with incomplete data; 2) Non-English language publications; 3) Trials terminated prematurely for various reasons.

Following initial study selection, a preliminary network graph was produced. In cases where key studies were missing, additional relevant studies were identified and included after thorough discussion, ensuring the completeness of the network graph. The study protocol was registered with PROSPERO (Registration number: CRD42023443943). Our approach to study selection and inclusion aligned with the Preferred Reporting Items for Systematic Reviews and Meta-Analyses (PRISMA) guidelines (8).

### Data sources and extraction

We conducted a comprehensive search of electronic databases, including PubMed and Web of Science, for studies published from their inception through April 3rd, 2023. The search included: 1) Completed Phase III or IV randomized controlled trials (RCTs) registered with ClinicalTrials.gov; 2) Patient cohorts with a confirmed diagnosis of metastatic castration-resistant prostate cancer (mCRPC) who had not previously received chemotherapy or novel endocrine therapies. The literature search employed the following terms, used as title/abstract keywords or MeSH terms: ‘castration-resistant prostate cancer’, ‘abiraterone’, ‘enzalutamide’, ‘docetaxel’, ‘Radium-223’, ‘cabazitaxel’.

Data extraction was independently conducted by two reviewers (ZD and WH), following a thorough assessment of all potential abstracts and titles for eligibility. In instances of disagreement or insufficient information, a third reviewer (MY) was consulted to examine the full text for eligibility. Extracted information included patient characteristics (median age, treatment descriptions, and doses) and sites of metastatic disease.

The analysis focused on median overall survival (OS) as the efficacy criterion, while toxicity criteria included the incidence of Grade 3-5 toxicities as per the National Cancer Institute Common Toxicity Criteria, along with the incidence of serious adverse events.

### Risk of bias assessment

Methodological quality of the included studies was assessed independently by two investigators, utilizing the Cochrane Handbook for Systematic Reviews of Interventions. Each trial was evaluated on the following criteria and assigned a risk of bias rating as low, medium, or high: random sequence generation, allocation concealment, blinding of participants and personnel, blinding of outcome assessment, completeness of outcome data, selective reporting, and presence of any other biases. A trial was deemed to have an overall low risk of bias if all domains were rated as low risk, and high risk if any domain was assessed as high risk. Discrepancies in assessment were resolved through discussion between the two investigators, or by consulting a third investigator for an adjudicated decision.

### Statistical analysis

The network meta-analysis was performed using a Bayesian framework model, employing R software (version 4.3.0) with the gemtc package (9). For the outcomes, overall survival (OS) was estimated using pooled hazard ratios (HRs) with 95% confidence intervals (CIs), and severe adverse events (SAEs) were analyzed using odds ratios (ORs) with 95% CIs. Both fixed-effects and random-effects models were fitted, with the latter accounting for heterogeneity between studies. The results presented in this study are based on the fixed-effects model.

## Results

### Study selection and network geometry

**Figure 1:**
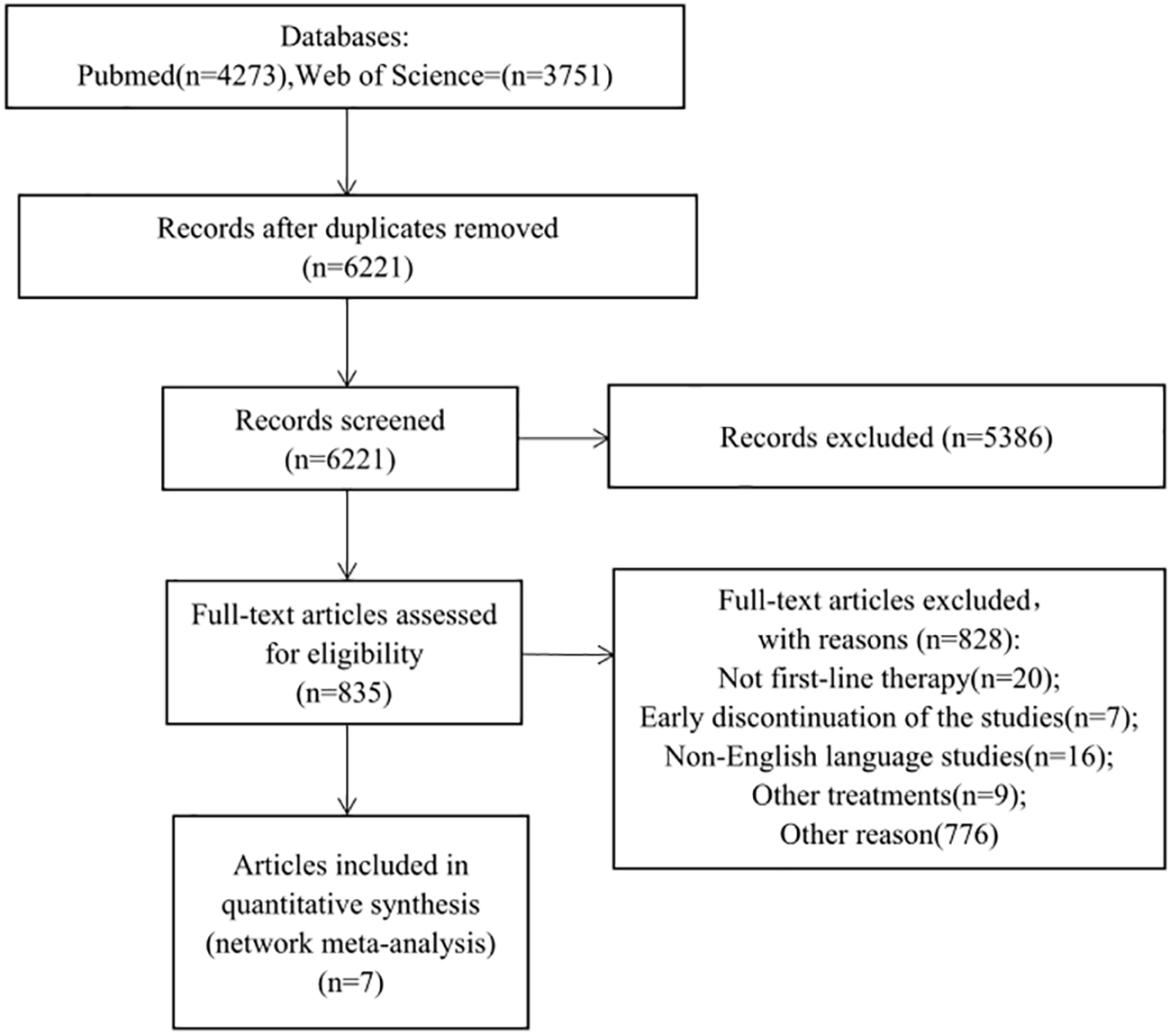
Study fow chart. This network meta-analysis incorporated 6 phase III and 1 phase IV randomized controlled trials (RCTs), enrolling a total of 6,411 patients with metastatic castration-resistant prostate cancer (mCRPC). Eight treatment modalities were analyzed: placebo/prednisone, abiraterone acetate + prednisone, enzalutamide, cabazitaxel 20/25mg/m^^2^, docetaxel, Radium-223 + abiraterone acetate + prednisone, and apalutamide + abiraterone acetate + prednisolone.

**Figure 2:**
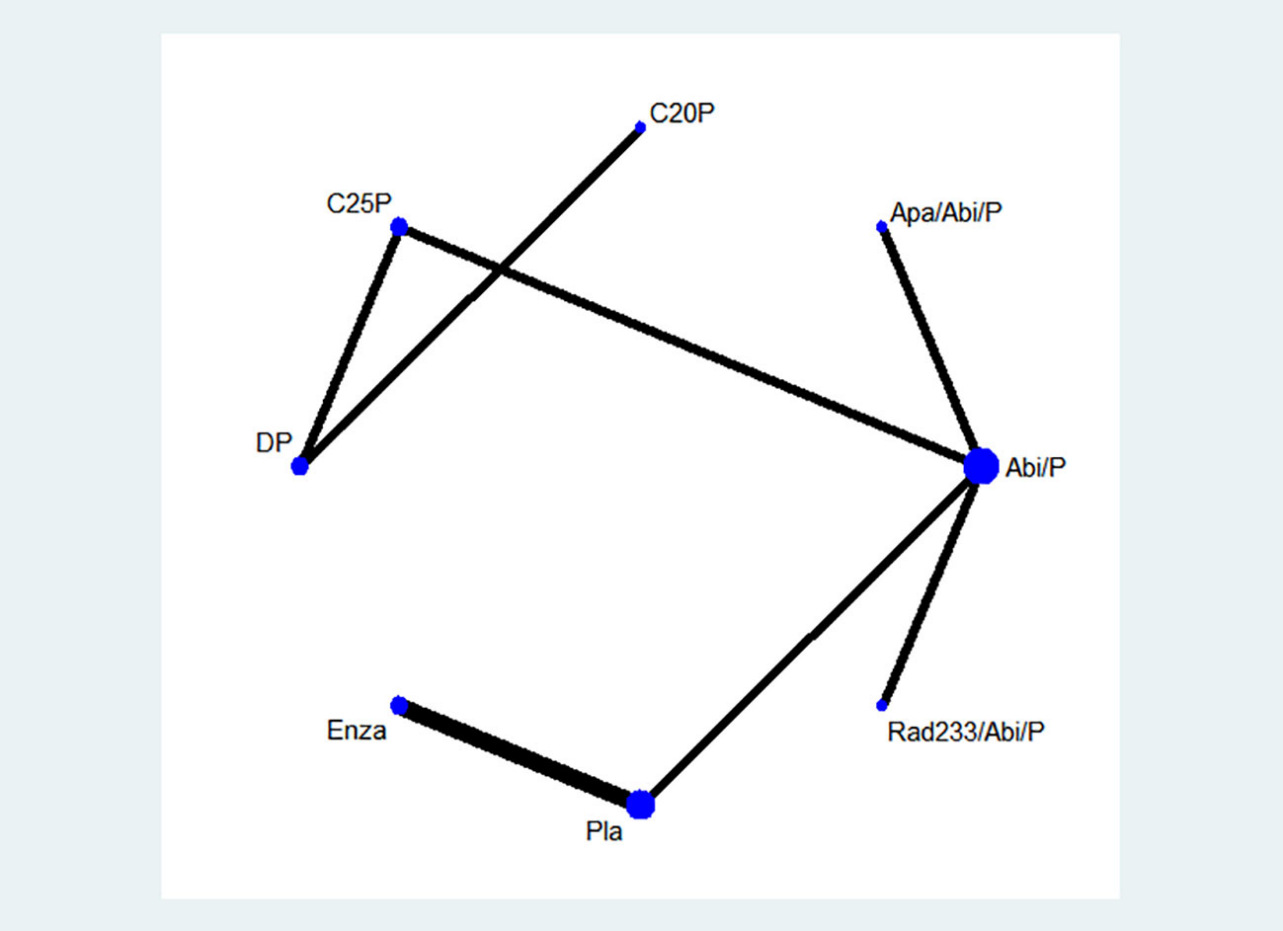
A network graph depicting treatment comparisons is illustrated.DP-docetaxel+prednisone. C25P-cabazitaxel 25 mg/m^^2^+prednisone, C20P-cabazitaxel 20 mg/m^^2^+prednisone, Apa/abi/p-apalutamide + abiraterone acetate + prednisolone, Abi/p-abiraterone acetate + prednisone, Rad233/abi/p-Radium-223 + abiraterone acetate + prednisone, Pla-placebo, Enza-enzalutamide.

Initial database screening yielded 4,273 references from PubMed and 3,751 from Web of Science (**Figure 1**). This was narrowed down to 835 potentially relevant trials after initial screening. Upon detailed examination, 7 studies fulfilling the inclusion criteria were selected for analysis. A network graph depicting treatment comparisons is illustrated in **Figure 2**.

The most frequently studied treatment was abiraterone acetate + prednisone (4 trials). To complete the network graph, a phase IV second-line treatment RCT comparing cabazitaxel 25mg/m^^2^ and abiraterone acetate was included after discussion.

### Characteristics of included trials

The analysis included seven multicenter RCTs, predominantly phase III first-line treatments, with the exception of one phase IV second-line treatment RCT included for network completeness (10–16). These trials spanned 2015 to 2020, involving a total of 6,411 participants. Median sample size per treatment arm was 396 (range, 126-872) patients; median age was 70.6 years (range, 68-71.6 years); median overall survival (OS) was 30.15 months (range, 11-39.1 months). Eligibility criteria primarily required newly diagnosed prostate adenocarcinoma with radiographic evidence of metastasis and adequate performance status, excluding or restricting prior chemotherapy and hormone therapy in the metastatic setting. To ensure network completeness, the CARD trial was additionally included. Baseline characteristics of the 7 studies are detailed in **Table 1**.

**Table 1:** Detailed description of baseline characteristics of 7 studies.

Trail	Study name, Registration	Patients enrolled	Treatment arms	Patients included for analysis	Median age(y)	Median OS(m)	No. of countries or areas
Charles J Ryan 2015	COU-AA-302	1088	Abiraterone Acetate + Prednisone	546	70.5	34.7	11 (Australia, Belgium, Canada, et al.)
	NCT00887198		Placebo+Prednisone	542	70.1	30.3	
Yohann Loriot 2015	PREVAIL	1717	Enzalutamide	872	71.3	32.4	23 (Australia, Austria, Belgium, et al.)
	NCT01212991		Placebo	845	71.2	30.2	
Stéphane Oudard 2017	FIRSTANA	1168	Cabazitaxel 20 mg/m^2	389	68.0	24.5	25 (Australia, Belarus, Brazil, et al.)
	NCT01308567		Docetaxel	391	69.0	24.3	
			Cabazitaxel 25 mg/m^2	388	68.5	25.2	
			Docetaxel	391	69.0	24.3	
Matthew Smith 2019	ERA 223	806	Radium-223 Dichloride + Abiraterone Acetate + Prednisone	401	70.9	30.1	19 (Australia, Belgium, Brazil, et al.)
	NCT02043678		Placebo+Abiraterone Acetate + Prednisone	405	71.4	34.8	
Karim Fizazi 2020	CARD	255	Cabazitaxel 25 mg/m^2	129	69.7	13.6	13 (Austria, Belgium, Czechia, et al.)
	NCT02485691		Abiraterone Acetate or Enzalutamide	126	69.7	11.0	
Fred Saad 2021	ACIS	982	Apalutamide + Abiraterone Acetate + Prednisolone	492	71.4	36.2	17 (Argentina, Australia, Belgium, et al.)
	NCT02257736		Placebo+ Abiraterone Acetate + Prednisolone	490	70.7	33.7	
Yeong-Shiau Pu 2022	9785-CL-0232	395	Enzalutamide	202	71.6	39.1	Asian
	NCT02294461		Placebo	193	71.0	27.1	

### Risk of bias

**Figure 3:**
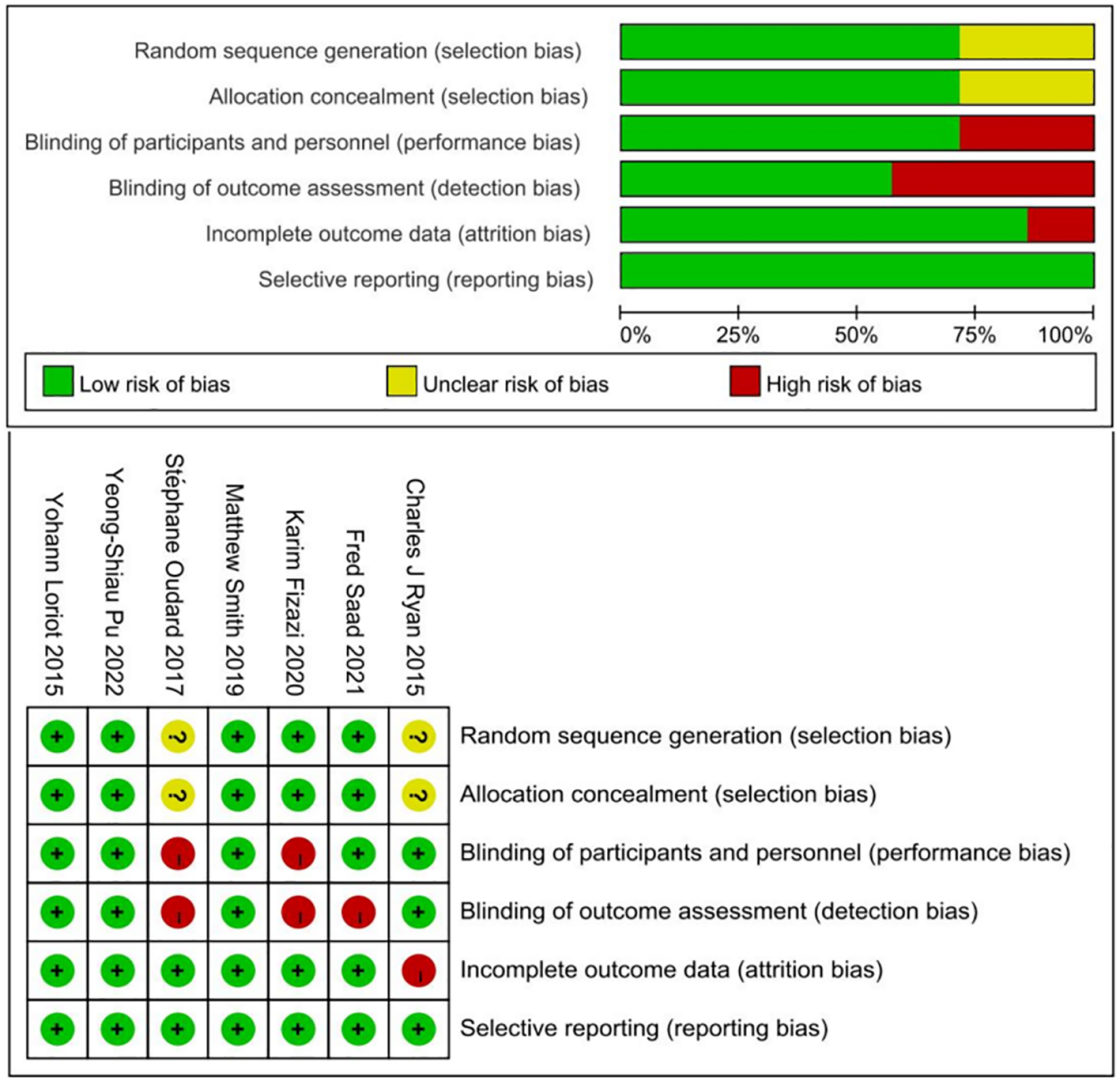
Risk of bias of selected studies.

The Cochrane Collaboration tool was employed for quality assessment of the included trials. Bias risk was evaluated across six domains mentioned in the selection criteria. Five of the seven studies demonstrated adequate randomization. The remaining studies lacked specific details on sequence generation methods. Allocation concealment was reported in five trials, with two trials employing open-label designs. Attrition and reporting biases were assessed and managed effectively in the included studies. A summary of the risk-of-bias assessment for each trial is presented in **Figure 3**.

### Syntheses of results

**Figure 4:**
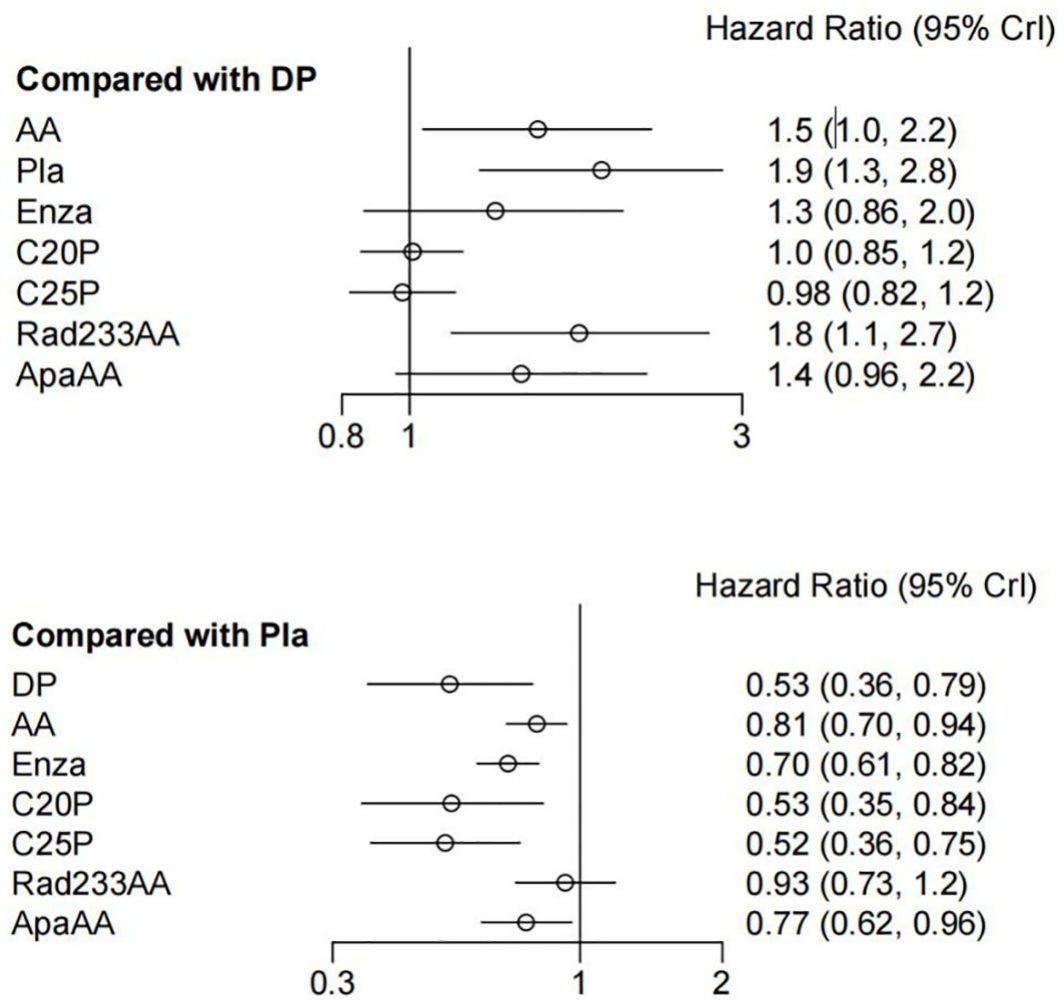
Summary of effectiveness (OS) and safety (SAE) assessments of eight treatments.

Network meta-analyses incorporated all eight treatments, evaluating both effectiveness (OS) and safety (SAEs) outcomes (**Figure 4**).

### Effectiveness outcomes

Treatments showing significant OS improvement over placebo included: docetaxel (HR, 0.53; 95% CI, 0.36-0.79), abiraterone acetate (HR, 0.81; 95% CI, 0.70-0.94), enzalutamide (HR, 0.70; 95% CI, 0.61-0.82), cabazitaxel 20 mg/m^^2^ (HR, 0.53; 95% CI, 0.35-0.84), and cabazitaxel 25 mg/m^^2^ (HR, 0.52; 95% CI, 0.36-0.75). Docetaxel also demonstrated superior OS improvement compared to abiraterone and Radium-223 + abiraterone, and was comparable with enzalutamide, cabazitaxel, and apalutamide (**Figure 4**). Treatment ranking probabilities indicated cabazitaxel 25 mg/m^^2^ as the most likely best treatment for OS (45% probability).

### Safety outcomes

Regarding SAEs, treatments ranked from safest to least safe were: docetaxel, cabazitaxel 20mg/m^^2^, cabazitaxel 25mg/m^^2^, abiraterone, Radium-223 combined with abiraterone, enzalutamide, and apalutamide combined with abiraterone. There were no significant differences between docetaxel, cabazitaxel 20/25 mg/m^^2^, and placebo in terms of SAEs.

### Heterogeneity

The heterogeneity of our findings (I^2^) was less than 30%, which indicated that our findings were homogeneous. Therefore, we did not conduct subgroup analysis to identify the source of heterogeneity. Comprehensive results can be found in **Supplementary Figure 3**.

### Grade assessment

Out of 7 RCTs, 4 of them were categorized as low risk of bias. Due to the lack of sufficient blinding methods, 3 RCTs were revealed to have high risk of bias. Based on grading the evidence in **Table 2**, 3 low risk of bias articles were included and produced high certainty of evidence. Based on the grading analysis, it is revealed that all 3 studies not only have low risk of bias but are also not serious in terms of inconsistency, indirectness, and imprecision. All these criteria increase the certainty level and can guide clinicians and policymakers for future events or discussions. Comprehensive results can be found in **Table 2**.

**Table 2:** Grading the evidence with GRADEpro Guideline Development Tool.

Certainty assessment							№ of patients		Effect	Certainty	Importance
№ of studies	Study design	Risk of bias	Inconsistency	Indirectness	Imprecision	Other considerations	Intervention	Control	Relative (95% CI)		
DP vs C20P(OS)											
1	randomised trials	serious	not serious	not serious	not serious	none	391 participants	389 participants	**HR 0.99**	⨁⨁⨁◯	CRITICAL
									(0.84 to 1.20)	Moderate
									[DP vs C20P(OS)]	
DP vs C25P(OS)											
1	randomised trials	not serious	not serious	serious	not serious	none	391 participants	388 participants	**HR 1.00**	⨁⨁⨁◯	CRITICAL
									(0.86 to 1.20)	Moderate
									[DP vs C25P(OS)]	
C25P vs Abi/P(OS)											
1	randomised trials	serious	not serious	serious	not serious	none	129 participants	126 participants	**HR 0.64**	⨁⨁◯◯	CRITICAL
									(0.46 to 0.89)	Low
									[C25P vs Abi/P(OS)]	
Abi/P vs Apa/Abi/P(OS)											
1	randomised trials	serious	not serious	not serious	not serious	none	492 participants	490 participants	**HR 1.10**	⨁⨁⨁◯	CRITICAL
									(0.89 to 1.20)	Moderate
									[Abi/P vs Apa/Abi/P(OS)]	
Abi/P vs Rad233/Abi/P(OS)											
1	randomised trials	not serious	not serious	not serious	not serious	none	405 participants	401 participants	**HR 0.87**	⨁⨁⨁⨁	CRITICAL
									(0.72 to 1.10)	High
									[Abi/P vs Rad233/Abi/P(OS)]	
Abi/P vs Pla(OS)											
1	randomised trials	not serious	not serious	not serious	not serious	none	546 participants	542 participants	**HR 0.81**	⨁⨁⨁⨁	CRITICAL
									(0.70 to 0.94)	High
									[Abi/P vs Pla(OS)]	
Enza vs Pla(OS)											
2	randomised trials	not serious	not serious	not serious	not serious	none	872 participants	845 participants	**HR 0.70**	⨁⨁⨁⨁	CRITICAL
									(0.61 to 0.82)	High
									[Enza vs Pla(OS)]	

CI, confidence interval; HR, hazard ratio; OR, odds ratio.

## Discussion

This network meta-analysis systematically evaluated first-line treatments for metastatic castration-resistant prostate cancer (mCRPC) as delineated in existing Phase III and IV randomized controlled trials (RCTs). A notable majority of these treatments had not previously been directly compared in face-to-face trials. Our comprehensive analysis revealed that chemotherapy regimens, specifically docetaxel and cabazitaxel, demonstrated superior efficacy and safety compared to second-generation anti-hormonal therapies, including abiraterone, enzalutamide, and apalutamide, in the first-line management of mCRPC.

The findings of this network meta-analysis provide new insights into the first-line treatment of metastatic castration-resistant prostate cancer (mCRPC). Current guidelines from the American Urological Association (AUA) recommend abiraterone and enzalutamide as grade A treatments, and docetaxel as a grade B treatment for mCRPC (17). Similarly, the Apccc expert consensus endorses abiraterone and enzalutamide as primary treatments (18). These recommendations contrast with our results, prompting an exploration of potential reasons for these discrepancies. Several factors may contribute to this variation:

AR-V7 Presence: AR-V7, a variant of the androgen receptor (AR) lacking the ligand-binding domain, is frequently observed in mCRPC patients, with about a 30% mutation rate. Antonarakis (19) demonstrated a significant correlation between AR-Vs in circulating tumor cells and clinical outcomes in CRPC patients receiving new AR-targeted therapies. Studies indicate that AR-V7 positivity is associated with resistance and poor efficacy in patients treated with enzalutamide and abiraterone (20). Conversely, AR-V7 status does not significantly affect responses to paclitaxel-based therapies like docetaxel or cabazitaxel (21).

PTEN Deficiency: The tumor suppressor gene PTEN, frequently lost or mutated in cancers, regulates the PI3K−AKT−mTOR signaling pathway. In mCRPC, PTEN gene deletion occurs in 40-60% of cases (22–24). Studies have shown that PTEN deficiency negatively impacts the effectiveness of abiraterone, but does not affect the antitumor activity of docetaxel (25, 26).

DDR Gene Mutations: The impact of DDR (DNA damage response) gene mutations on second-generation hormone therapy and paclitaxel-based therapy remains unclear. Some studies suggest that DDR gene mutations attenuate the efficacy of second-generation hormone therapies, but their impact on the efficacy of cabazitaxel is less certain (27, 28).

Tumor Neuroendocrine Differentiation (NED): NED in mCRPC is a significant factor in treatment response. Hormone therapy is generally less effective in patients with NED. A study found greater OS benefit with a Docetaxel+Prednisone (DP) - Abiraterone Acetate (AA) treatment sequence in patients with elevated NED, compared to an AA-DP sequence (29).In light of these findings, our analysis suggests that the choice of first-line treatment for mCRPC should consider molecular and genetic tumor characteristics to optimize patient outcomes.

The objective of this network meta-analysis was to elucidate the efficacy of cabazitaxel 20 mg/m^^2^ (C20P) and cabazitaxel 25 mg/m^^2^ (C25P) over docetaxel+prednisone (DP) in chemotherapy- or hormone therapy-naive patients with metastatic castration-resistant prostate cancer (mCRPC), focusing on overall survival (OS). Our recommendation favors C20P, as it shows comparable OS to C25P but exhibits superior safety in terms of severe adverse events (SAEs), suggesting enhanced tolerability at the lower dose.

The choice of optimal treatment for mCRPC remains a subject of debate. Recently, some scholars have also performed network meta-analysis for first-line treatment of mCRPC (30). Unlike our analysis, this analysis included all period RCTs to form a network and was therefore more exploratory. Our analysis includes Phase III and IV RCTs, and focuses on the high level of evidence to guide clinical application. This analysis included 29 RCTs, involving 12,706 patients and investigating 16 interventions. According to the OS results of this analysis, in addition to docetaxel and cabazitaxel-based chemotherapy regimens, chemotherapy combined with targeted therapy (capivasertib or cabozantinib) and chemotherapy combined with PD-1 (ipilimumab) showed significant effects. Cabozantinib is a tyrosine kinase inhibitor that targets multiple genes, including MET, VEGFR1/2/3, ROS1, RET, AXL, NTRK, and KIT, and is currently used to treat renal cancer (31). Capivasertib is an AKT inhibitor with potential efficacy in patients with PIK3CA, AKT1, and or PTEN mutations. These therapeutic strategies of targeted therapy combined with chemotherapy are providing a new direction for mCRPC.

Different levels of genetic mutations are often found in malignant tumors, which can cause poor response to castration therapy or chemotherapy. Therefore, the use of monotherapy in cancer therapy has limitations, and monotherapy specifically inhibits a therapeutic target and triggers compensatory mechanisms or other signal transduction bypasses, which require the assistance of other drugs to improve efficacy. Researchers are increasingly interested in using combinations of low-dose anti-cancer agents with different modes of action rather than administering single agents at high doses. Combinations of anticancer drugs with different mechanisms of action may show synergistic effects in inhibiting the growth of prostate cancer cells and inducing apoptosis. In response to the aberrant activation of PI3K and NF-κB pathways in the late stage of docetaxel chemotherapy in mCRPC, drugs that can inhibit the transduction of this signaling pathway are required.

In order to achieve precise treatment of mCRPC, genetic testing of patients with mCRPC is required. For patients who have undergone surgical treatment, tumor specimens can be sampled and tested. For inoperable patients, prostate biopsy may be used for testing. Both approaches can be applied to most scenarios. In addition, circulating tumor cells (CTC) can be used to detect novel mutations. CTCs are tumor cells disseminated from primary and/or metastatic tumor sites that circulate in the vasculature with potential for distant seeding. Studies have shown that CTC detection has been performed in mCRPC patients by a useful platform to detect the presence or absence of AR-V7 mutations (32). A multicentric study replicated these findings using an open-source Automated CTC Classification Enumeration and PhenoTyping software for the prognostication of mCRPC patients (33). In addition to CTC, mCRPC can also be genetically classified by detecting circulating nucleic acids, extracellular vesicles. After that targeted or immune treatment regimens can be used for different types mutations to achieve precise treatment of mCRPC.

According to our analysis, cabazitaxel or docetaxel is preferable over abiraterone or enzalutamide for initial chemotherapy or hormone therapy in mCRPC patients who have not undergone genetic testing. Moreover, there is considerable potential for advancement in prostate cancer treatment. The efficacy of many therapeutics is closely linked to tumor genetic mutations, indicating a need for further research in this area.

## Limitations

Our study is not without limitations. Firstly, due to exclusion criteria, we were unable to include emerging treatments such as targeted therapies (olaparib, ipatasertib), vaccine therapies (sipuleucel-T), and radiation therapy (177Lu-PSMA-617). Secondly, the inclusion of a second-line treatment RCT for mCRPC (CARD) was necessary to complete the network graph, which may have introduced bias. It is hoped that future analyses will incorporate more Phase III RCTs focused on first-line mCRPC treatments. Thirdly, the field of prostate cancer treatment is yet to fully embrace precision therapy, and many studies lack genetic data. Therefore, a subgroup analysis of genetic factors in the included patients was not feasible.

## Conclusions

We recommend cabazitaxel 20 mg/m^^2^ as the primary option for first-line treatment of mCRPC. Genetic testing for mCRPC patients is also advised to tailor treatment choices based on mutation profiles. Given the limitations of our network meta-analysis, the need for more comprehensive, high-quality studies for further evaluation is evident.

## Data availability statement

The raw data supporting the conclusions of this article will be made available by the authors, without undue reservation.

## Author contributions

DZ: Writing – original draft, Investigation, Data curation, Conceptualization. HW: Writing – original draft, Investigation, Data curation, Conceptualization. ZZ: Writing – original draft, Data curation, Conceptualization. WG: Writing – original draft, Data curation, Conceptualization. YM: Writing – review & editing, Supervision, Project administration, Investigation, Funding acquisition, Writing – original draft, Data curation, Conceptualization.
