# Supplementary material for: Evaluating first-line therapeutic strategies for metastatic castration-resistant prostate cancer: a comprehensive network meta-analysis and systematic review
Source: Front Oncol. 2024 Apr 15;14:1378993. doi: 10.3389/fonc.2024.1378993 (PMC11056588; doi:10.3389/fonc.2024.1378993)
Supplement: Supplementary file 1 [file DataSheet_1.doc]

**Supplementary Online Content**

**eMethods.1** Search Strategies

**eTable.1** Relative Effect Estimates for Included First-line Treatment Comparisons

**eTable.2** Rank Probability for Overall Survival and Serious Adverse Events

**eTable.3** Cumulative Ranking Probability(CUCRA) for Overall Survival and Serious Adve**rse Events**

**eFig.1** Forest plot for Overall Survival and Serious Adverse Events

**eFig.2** Treatment Ranking Probabilities for Overall Survival and Serious Adverse Events

**eFig.3** Plot for Heterogeneity Test

**eMethods 1.** Search Strategies

PubMed

| #1 | (((((((((Prostatic Neoplasms[Mesh]) OR (Prostatic Neoplasms[Text Word])) OR (Prostate Neoplasm[Text Word])) OR (Prostatic Neoplasm[Text Word])) OR (Prostate Cancer[Text Word])) OR (Prostate Cancers[Text Word])) OR (Cancer of the Prostate[Text Word])) OR (Prostatic Cancer[Text Word])) OR (Prostatic Cancers[Text Word])) OR (Cancer of Prostate[Text Word]) |
| --- | --- |
| #2 | ((((enzalutamide[Supplementary Concept]) OR (HC 1119[Text Word])) OR (Xtandi[Text Word])) OR (MDV 3100[Text Word])) OR (enzalutamide[Text Word]) |
| #3 | (((Abiraterone Acetate[Supplementary Concept]) OR (Zytiga[Text Word])) OR (CB7630[Text Word])) OR (abiraterone[Text Word]) |
| #4 | (((((((((((Docetaxel Trihydrate[Text Word]) OR (Docetaxol[Text Word])) OR (Docetaxel Hydrate[Text Word])) OR (Taxoltere Metro[Text Word])) OR (RP 56976[Text Word])) OR (Taxotere[Text Word])) OR (Docetaxel Anhydrous[Text Word])) OR (N-Debenzoyl-N-tert-butoxycarbonyl-10-deacetyltaxol[Text Word])) OR (N Debenzoyl N tert butoxycarbonyl 10 deacetyltaxol[Text Word])) OR (NSC 628503[Text Word])) OR (Docetaxel[MeSH])) OR (Docetaxel[Text Word]) |
| #5 | (((olaparib[Supplementary Concept]) OR (olaparib[Text Word])) OR (AZD2281[Text Word])) OR (Lynparza[Text Word]) |
| #6 | ((177Lu-PSMA-617[Supplementary Concept]) OR (177Lu-PSMA-617[Text Word])) OR (177Lu-617-prostate-specific membrane antigen ligand[Text Word]) |
| #7 | (((Radium-223 [Supplementary Concept]) OR (Radium-223[Text Word])) OR (Ra-223 radioisotope[Text Word])) OR (223Ra radioisotope[Text Word]) |
| #8 | (((((sipuleucel-T [Supplementary Concept])OR (sipuleucel-T[Text Word])) OR (APC 8015[Text Word])) OR (APC8015[Text Word])) OR (APC-8015[Text Word])) OR (Provenge[Text Word]) |
| #9 | (((cabazitaxel [Supplementary Concept]) OR (cabazitaxel[Text Word])) OR (kabazitaxel[Text Word])) OR (Jevtana[Text Word]) |
| #10 | ((((ipatasertib [Supplementary Concept]) OR (ipatasertib[Text Word])) OR (2-(4-chlorophenyl)-1-(4-(7-hydroxy-5-methyl-6,7-dihydro-5H-cyclopenta(d)pyrimidin-4-yl)piperazin-1-yl)-3-(isopropylamino)propan-1-one[Text Word])) OR (GDC-0068[Text Word])) OR (GDC0068[Text Word]) |
| #11 | ((((Castration Resistant[Text Word]) OR (Androgen Insensitive[Text Word])) OR (Androgen Resistant[Text Word])) OR (Hormone Refractory[Text Word])) OR (Androgen Independent[Text Word]) |
| #12 | ((randomized controlled trial[pt]) OR (controlled clinical trial[pt]) OR (randomised[tiab] OR randomized[tiab]) OR (placebo[tiab]) OR (drug therapy[sh]) OR (randomly[tiab]) OR (trial[tiab]) OR (groups[tiab])) NOT (animals[mh] NOT humans[mh]) |
| #13 | #2 OR #3 OR #4 OR #5 OR #6 OR #7 OR #8 OR #9 OR #10 |
| #14 | #1 AND #11 |
| #15 | #13 AND #14 AND #12 |

Web of science

| #1 | (((((((((((((((((TI=(Prostatic Neoplasms)) OR TI=(Prostate Neoplasm)) OR TI=(Prostatic Neoplasm)) OR TI=(Prostate Cancer)) OR TI=(Prostate Cancers)) OR TI=(Cancer of the Prostate)) OR TI=(Prostatic Cancer)) OR TI=(Prostatic Cancers)) OR TI=(Cancer of Prostate)) OR AB=(Prostatic Neoplasms)) OR AB=(Prostate Neoplasm)) OR AB=(Prostatic Neoplasm)) OR AB=(Prostate Cancer)) OR AB=(Prostate Cancers)) OR AB=(Cancer of the Prostate)) OR AB=(Prostatic Cancer)) OR AB=(Prostatic Cancers)) OR AB=(Cancer of Prostate) |
| --- | --- |
| #2 | (((((((TI=(enzalutamide)) OR TI=(HC 1119)) OR TI=(Xtandi)) OR TI=(MDV 3100)) OR AB=(enzalutamide)) OR AB=(HC 1119)) OR AB=(Xtandi)) OR AB=(MDV 3100) |
| #3 | (((((TI=(Abiraterone Acetate)) OR TI=(Zytiga)) OR TI=(CB7630)) OR AB=(Abiraterone Acetate)) OR AB=(Zytiga)) OR AB=(CB7630) |
| #4 | (((((((((((((((((((((TI=(Docetaxel Trihydrate)) OR TI=(Docetaxol)) OR TI=(Docetaxel Hydrate)) OR TI=(Taxoltere Metro)) OR TI=(RP 56976)) OR TI=(Taxotere)) OR TI=(Docetaxel Anhydrous)) OR TI=(N-Debenzoyl-N-tert-butoxycarbonyl-10-deacetyltaxol)) OR TI=(N Debenzoyl N tert butoxycarbonyl 10 deacetyltaxol)) OR TI=(NSC 628503)) OR TI=(Docetaxel)) OR AB=(Docetaxel)) OR AB=(Docetaxel Trihydrate)) OR AB=(Docetaxol)) OR AB=(Docetaxel Hydrate)) OR AB=(Taxoltere Metro)) OR AB=(RP 56976)) OR AB=(Taxotere)) OR AB=(Docetaxel Anhydrous)) OR AB=(N-Debenzoyl-N-tert-butoxycarbonyl-10-deacetyltaxol)) OR AB=(N Debenzoyl N tert butoxycarbonyl 10 deacetyltaxol)) OR AB=(NSC 628503) |
| #5 | (((((TI=(olaparib)) OR TI=(AZD2281)) OR TI=(Lynparza)) OR AB=(olaparib)) OR AB=(AZD2281)) OR AB=(Lynparza) |
| #6 | (((TI=(177Lu-PSMA-617)) OR TI=(177Lu-617-prostate-specific membrane antigen ligand)) OR AB=(177Lu-617-prostate-specific membrane antigen ligand)) OR AB=(177Lu-PSMA-617) |
| #7 | (((((TI=(Radium-223)) OR TI=(Ra-223 radioisotope)) OR TI=(223Ra radioisotope)) OR AB=(Radium-223)) OR AB=(Ra-223 radioisotope)) OR AB=(223Ra radioisotope) |
| #8 | (((((((((TI=(sipuleucel-T)) OR TI=(APC 8015)) OR TI=(APC8015)) OR TI=(APC-8015)) OR TI=(Provenge)) OR AB=(sipuleucel-T)) OR AB=(APC 8015)) OR AB=(APC8015)) OR AB=(APC-8015)) OR AB=(Provenge) |
| #9 | (((((TI=(cabazitaxel)) OR TI=(kabazitaxel)) OR TI=(Jevtana)) OR AB=(cabazitaxel)) OR AB=(kabazitaxel)) OR AB=(Jevtana) |
| #10 | (((((((TI=(ipatasertib)) OR TI=(2-(4-chlorophenyl)-1-(4-(7-hydroxy-5-methyl-6,7-dihydro-5H-cyclopenta(d)pyrimidin-4-yl)piperazin-1-yl)-3-(isopropylamino)propan-1-one)) OR TI=(GDC-0068)) OR TI=(GDC0068)) OR AB=(ipatasertib)) OR AB=(2-(4-chlorophenyl)-1-(4-(7-hydroxy-5-methyl-6,7-dihydro-5H-cyclopenta(d)pyrimidin-4-yl)piperazin-1-yl)-3-(isopropylamino)propan-1-one)) OR AB=(GDC-0068)) OR AB=(GDC0068) |
| #11 | (((((((((TI=(Castration Resistant)) OR TI=(Androgen Insensitive)) OR TI=(Androgen Resistant)) OR TI=(Hormone Refractory)) OR TI=(Androgen Independent)) OR AB=(Castration Resistant)) OR AB=(Androgen Insensitive)) OR AB=(Androgen Resistant)) OR AB=(Hormone Refractory)) OR AB=(Androgen Independent) |
| #12 | ((((((((((TS=(clinical trial)) OR TS=(research design)) OR TS=(comparative stud*)) OR TS=(evaluation stud*)) OR TS=(controlled trial*)) OR TS=(follow-up stud*)) OR TS=(prospective stud*)) OR TS=(random*)) OR TS=(placebo*)) OR TS=(single blind*)) OR TS=(double blind*) |
| #13 | #2 OR #3 OR #4 OR #5 OR #6 OR #7 OR #8 OR #9 OR #10 |
| #14 | #1 AND #11 |
| #15 | #13 AND #14 AND #12 |

**eTable.1 Relative Effect Estimates for Included First-line Treatment Comparisons**

**Overall survival, hazard ratio (95% CI)**

| DP |  |  |  |  |  |  |  |
| --- | --- | --- | --- | --- | --- | --- | --- |
| -0.42 (-0.79, -0.05) | AA |  |  |  |  |  |  |
| -0.63 (-1.03, -0.23) | -0.21 (-0.36, -0.06) | Pla |  |  |  |  |  |
| -0.28 (-0.70, 0.15) | 0.14 (-0.07, 0.35) | 0.35 (0.20, 0.50) | Enza |  |  |  |  |
| -0.01 (-0.18, 0.16) | 0.41 (0.00, 0.82) | 0.62 (0.18, 1.06) | 0.27 (-0.19, 0.73) | C20P |  |  |  |
| 0.02 (-0.15, 0.20) | 0.44 (0.11, 0.77) | 0.66 (0.29, 1.01) | 0.30 (-0.09, 0.69) | 0.03 (-0.20, 0.28) | C25P |  |  |
| -0.56 (-0.98, -0.14) | -0.14 (-0.34, 0.06) | 0.07 (-0.18, 0.31) | -0.28 (-0.57, 0.00) | -0.55 (-1.00, -0.10) | -0.58 (-0.97, -0.20) | Rad233AA |  |
| -0.37 (-0.76, 0.03) | 0.05 (-0.11, 0.21) | 0.26 (0.05, 0.48) | -0.09 (-0.35, 0.17) | -0.36 (-0.82, 0.09) | -0.40 (-0.76, -0.03) | 0.19 (-0.06, 0.45) | ApaAA |

**Serious adverse events, odds ratio (95% CI)**

| DP |  |  |  |  |  |  |  |
| --- | --- | --- | --- | --- | --- | --- | --- |
| -0.72 (-1.63, 0.23) | AA |  |  |  |  |  |  |
| -0.23 (-1.17, 0.76) | 0.50 (0.25, 0.76) | Pla |  |  |  |  |  |
| -0.94 (-1.90, 0.06) | -0.21 (-0.52, 0.09) | -0.72 (-0.89, -0.53) | Enza |  |  |  |  |
| -0.08 (-0.38, 0.23) | 0.64 (-0.36, 1.60) | 0.14 (-0.89, 1.14) | 0.86 (-0.19, 1.86) | C20P |  |  |  |
| -0.66 (-0.94, -0.36) | 0.07 (-0.83, 0.93) | -0.43 (-1.37, 0.46) | 0.28 (-0.66, 1.20) | -0.57 (-1.00, -0.16) | C25P |  |  |
| -0.78 (-1.74, 0.21) | -0.06 (-0.34, 0.22) | -0.56 (-0.93, -0.18) | 0.16 (-0.25, 0.57) | -0.70 (-1.70, 0.35) | -0.12 (-1.03, 0.81) | Rad233AA |  |
| -0.94 (-1.91, 0.03) | -0.22 (-0.49, 0.04) | -0.72 (-1.09, -0.36) | -0.01 (-0.41, 0.39) | -0.86 (-1.87, 0.16) | -0.28 (-1.20, 0.64) | -0.17 (-0.55, 0.22) | ApaAA |

**eTable.2 Rank Probability for Overall Survival and Serious Adverse Events**

**Overall survival**

|  | 1 | 2 | 3 | 4 | 5 | 6 | 7 | 8 |
| --- | --- | --- | --- | --- | --- | --- | --- | --- |
| AA | 0.000125 | 0.000500 | 0.005250 | 0.032125 | 0.287375 | 0.621500 | 0.053000 | 0.000125 |
| ApaAA | 0.007500 | 0.013875 | 0.023625 | 0.233500 | 0.463875 | 0.204125 | 0.050000 | 0.003500 |
| C20P | 0.284375 | 0.244250 | 0.361625 | 0.065625 | 0.023125 | 0.013000 | 0.006500 | 0.001500 |
| C25P | 0.454375 | 0.247750 | 0.273625 | 0.018875 | 0.004000 | 0.001000 | 0.000375 | 0.000000 |
| DP | 0.209625 | 0.449000 | 0.282500 | 0.039375 | 0.011500 | 0.006250 | 0.001625 | 0.000125 |
| Enza | 0.044000 | 0.044125 | 0.052375 | 0.600250 | 0.172250 | 0.068500 | 0.018500 | 0.000000 |
| Pla | 0.000000 | 0.000000 | 0.000000 | 0.000000 | 0.001250 | 0.011000 | 0.273875 | 0.713875 |
| Rad233AA | 0.000000 | 0.000500 | 0.001000 | 0.010250 | 0.036625 | 0.074625 | 0.596125 | 0.280875 |

**Serious adverse events**

|  | 1 | 2 | 3 | 4 | 5 | 6 | 7 | 8 |
| --- | --- | --- | --- | --- | --- | --- | --- | --- |
| AA | 0.000625 | 0.027500 | 0.226125 | 0.450625 | 0.211250 | 0.048500 | 0.035375 | 0.000000 |
| ApaAA | 0.369375 | 0.345750 | 0.172875 | 0.071000 | 0.031750 | 0.006500 | 0.002750 | 0.000000 |
| C20P | 0.000625 | 0.031625 | 0.024500 | 0.029250 | 0.028625 | 0.215875 | 0.451125 | 0.217375 |
| C25P | 0.206375 | 0.097500 | 0.094125 | 0.076625 | 0.347625 | 0.177625 | 0.000125 | 0.000000 |
| DP | 0.000000 | 0.005750 | 0.017000 | 0.017375 | 0.022375 | 0.091125 | 0.376750 | 0.469625 |
| Enza | 0.353125 | 0.314500 | 0.184875 | 0.089875 | 0.039375 | 0.012750 | 0.005500 | 0.000000 |
| Pla | 0.000000 | 0.000000 | 0.000000 | 0.000375 | 0.176500 | 0.412000 | 0.099750 | 0.311375 |
| Rad233AA | 0.069875 | 0.177375 | 0.280500 | 0.264875 | 0.142500 | 0.035625 | 0.028625 | 0.000625 |

**eTable.3 Cumulative Ranking Probability(CUCRA) for Overall Survival and Serious Adverse Events**

**Overall survival**

| AA | ApaAA | C20P | C25P |
| --- | --- | --- | --- |
| 0.3309643 | 0.4339643 | 0.8040893 | 0.8750179 |
| DP | Enza | Pla | Rad233AA |
| 0.8257143 | 0.5582679 | 0.0428036 | 0.1291786 |

**Serious adverse events**

| AA | ApaAA | C20P | C25P |
| --- | --- | --- | --- |
| 0.5526607 | 0.8456429 | 0.2003393 | 0.6007143 |
| DP | Enza | Pla | Rad233AA |
| 0.1164464 | 0.8274107 | 0.2078214 | 0.6489643 |

**eFig.1 Forest plot for Overall Survival and Serious Adverse Events**

**Overall survival**


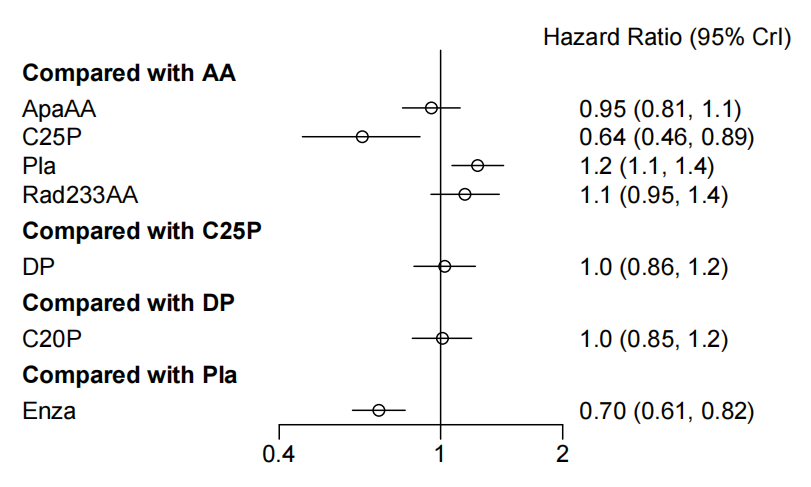


**
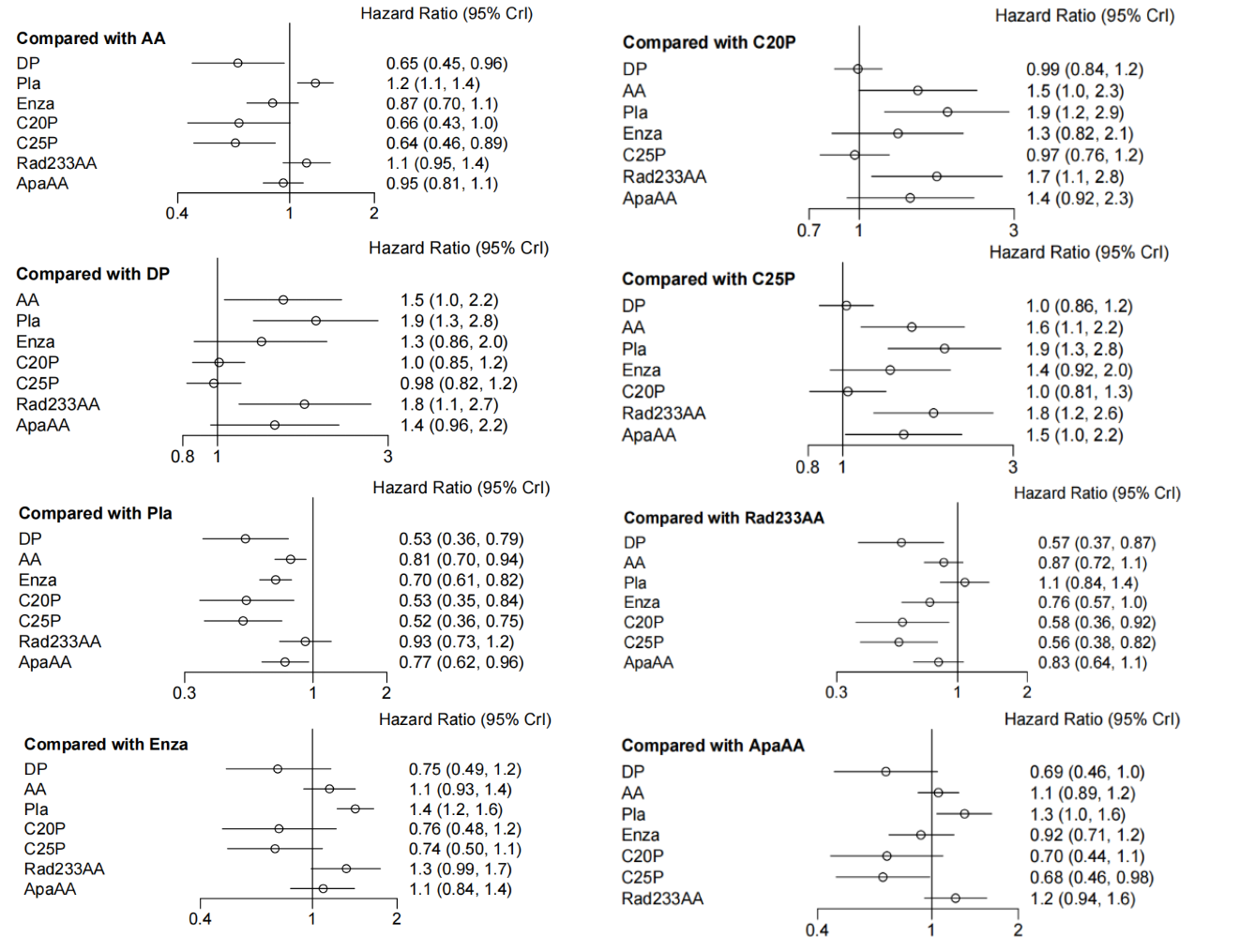
**

**Serious adverse events**


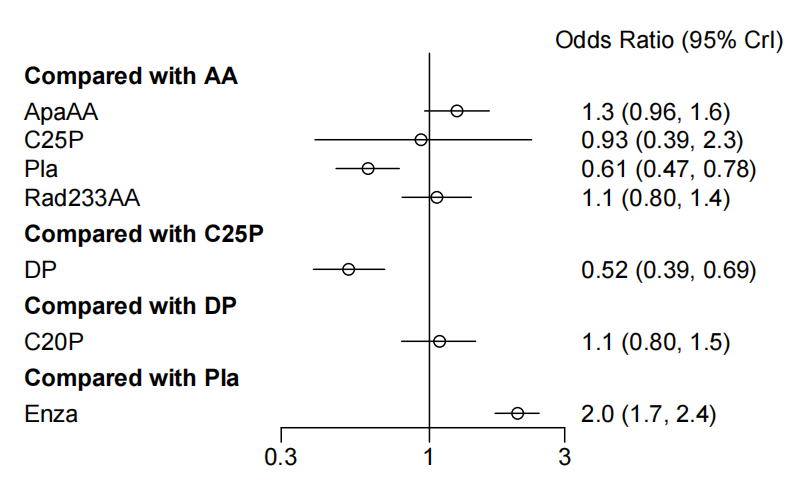


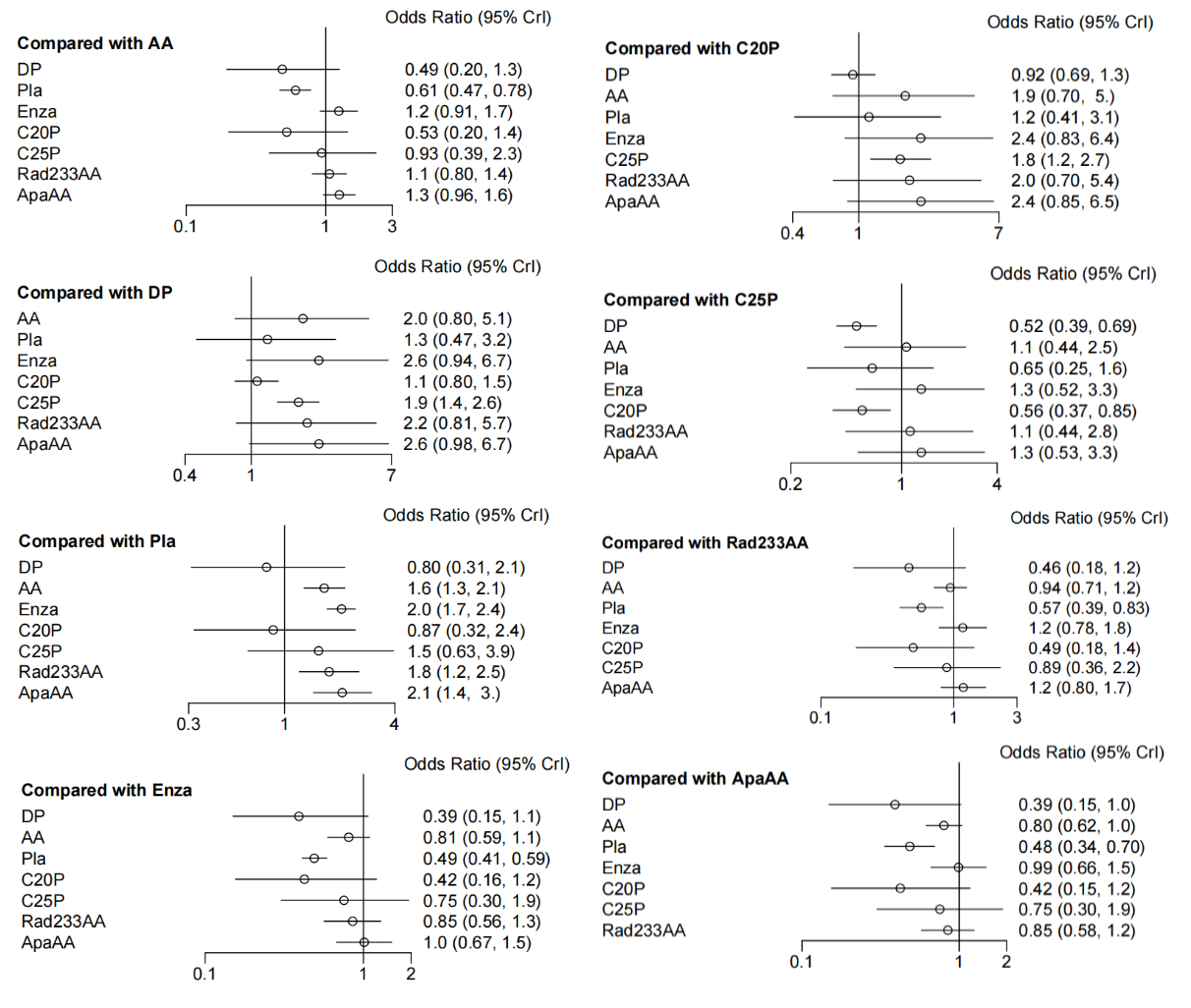


**eFig.2 Treatment Ranking Probabilities for Overall Survival and Serious Adverse Events**

**Overall survival**


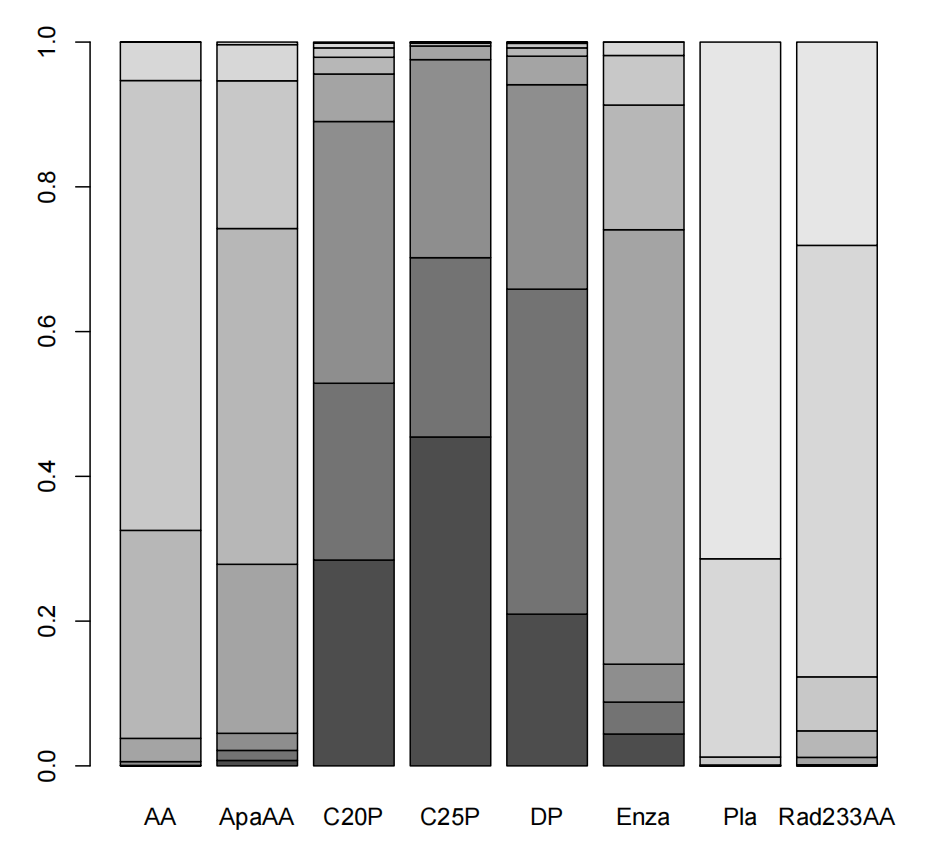


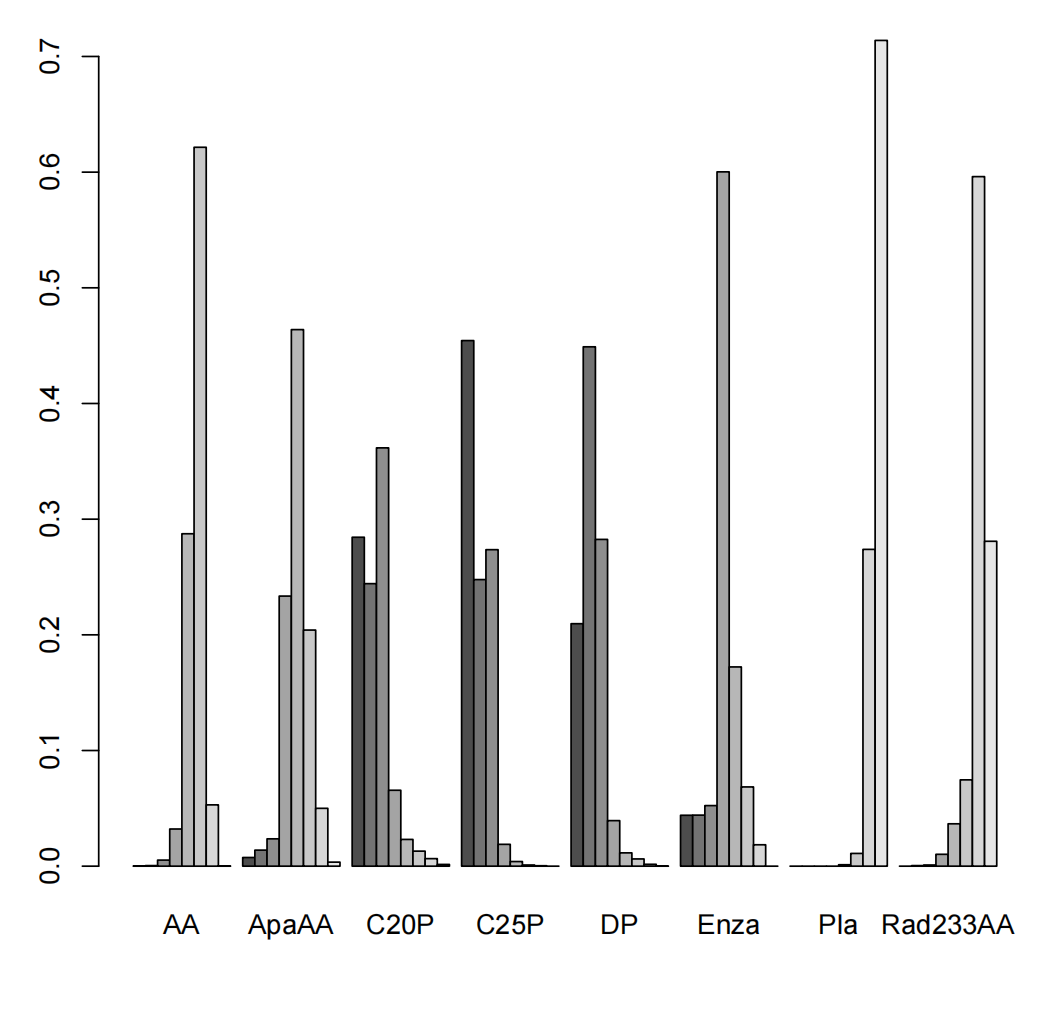


**Serious adverse events**


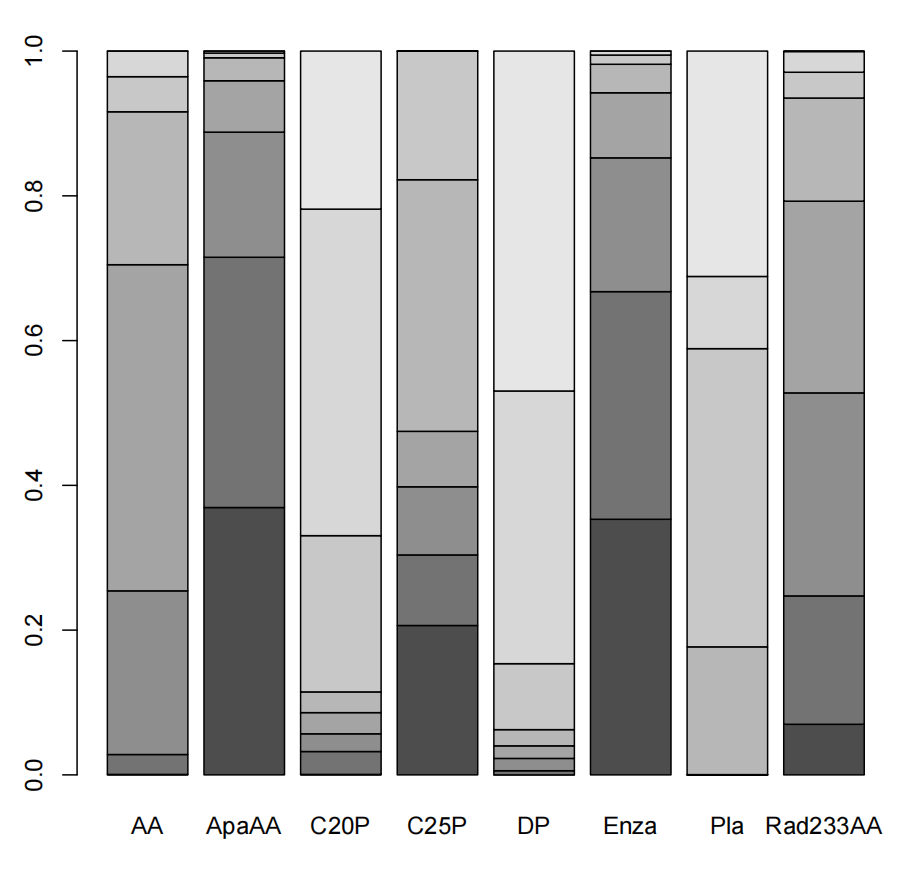


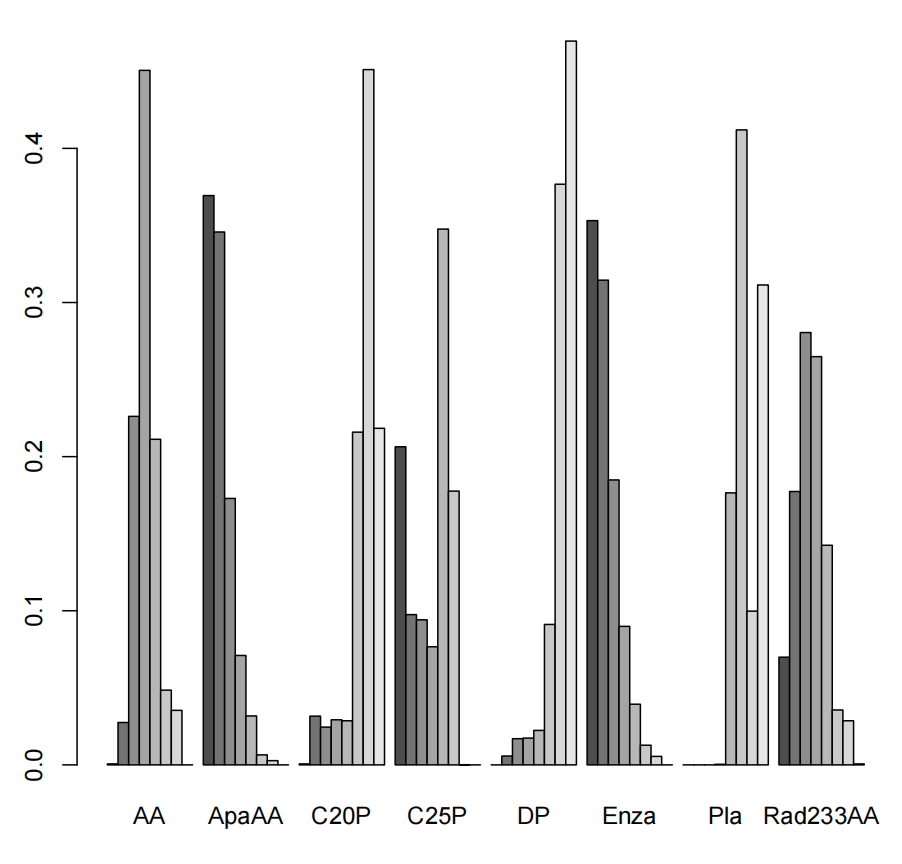


**eFig.3 Plot for Heterogeneity Test**

**
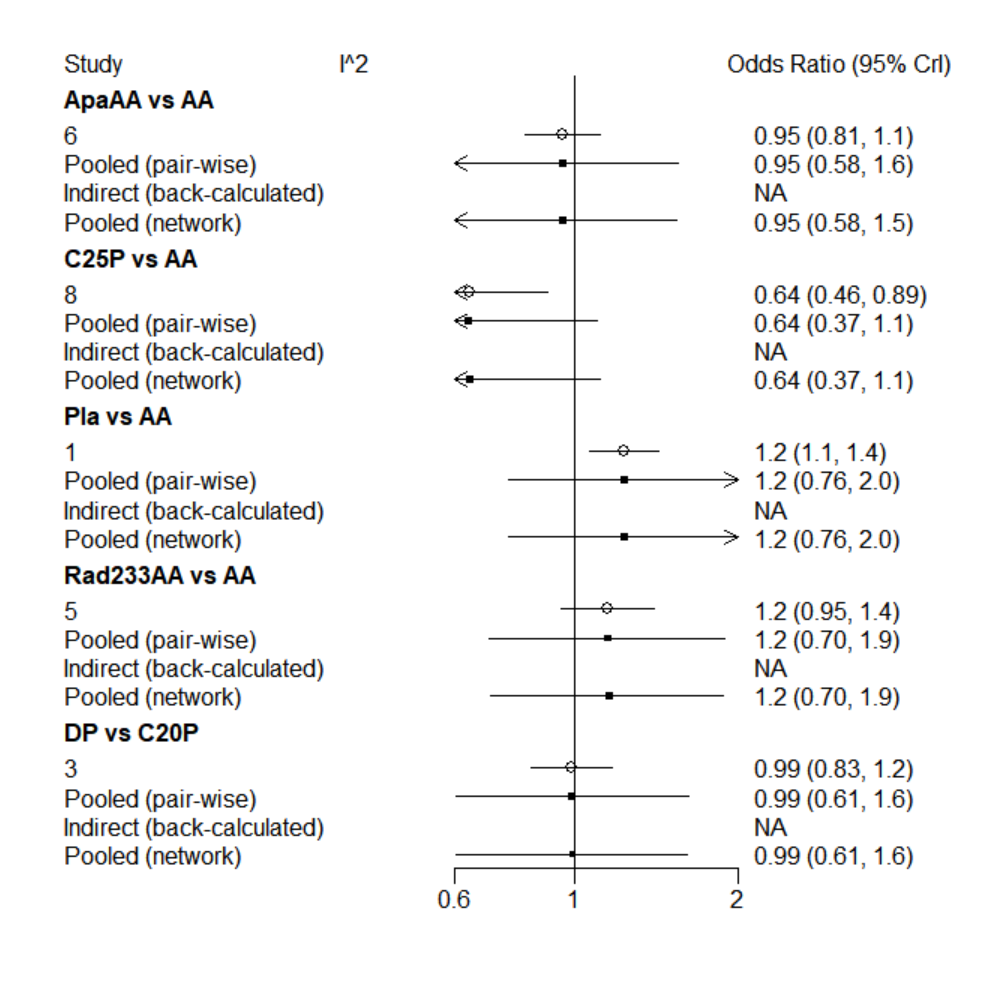

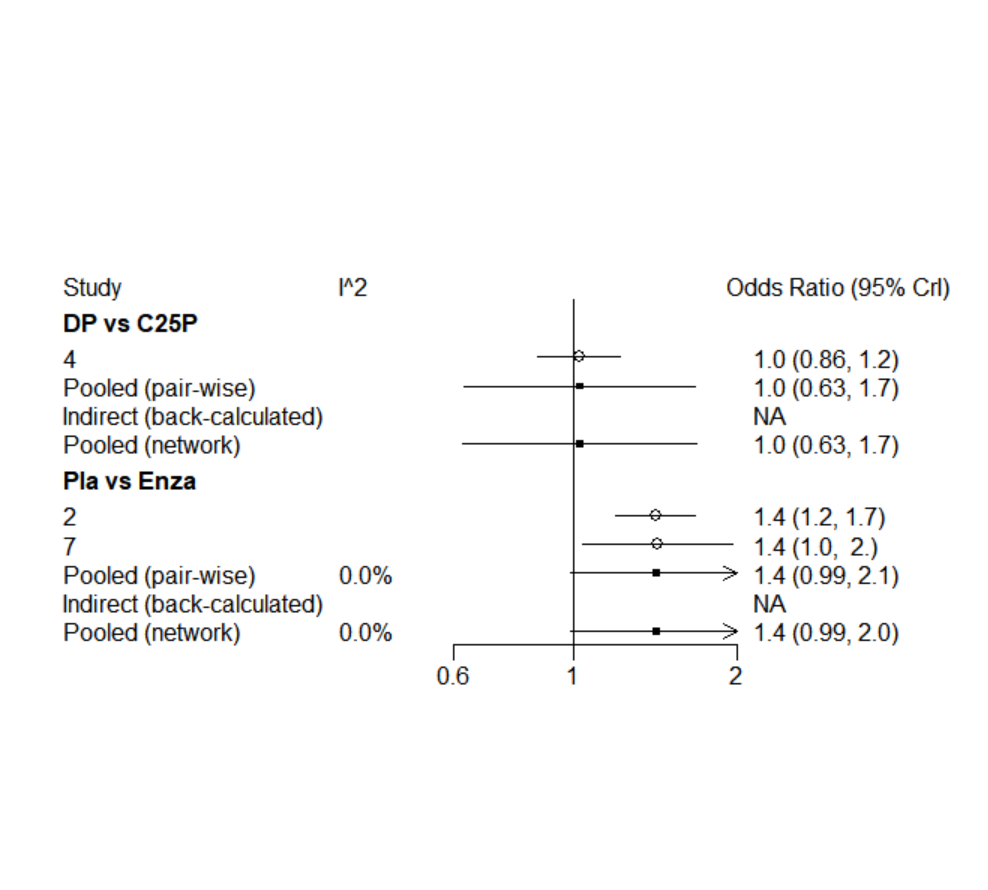
**
